# Supplementary material for: Construction and Validation of Nursing Actions to Integrate Mobile Care–Educational Technology to Assist Individual in Psychic Distress
Source: Int J Environ Res Public Health. 2025 Mar 13;22(3):419. doi: 10.3390/ijerph22030419 (PMC11941836; doi:10.3390/ijerph22030419)
Supplement: Supplementary file 1 [file ijerph-22-00419-s001.zip › Additional files-Table S6-Nursing actions for individuals in psychological distress use of psychoactive substances.pdf]

Table S6- Nursing actions for individuals in psychological distress: use of psychoactive substances and situations of mourning.

Categories and items	1st Round		Items<br>Changes and/or Additions	2nd Round	
	CVI (%)	α		CVI (%)	α
**E- Nursing Actions towards Persons in Psychic Distress resulting from the Use of Psychoactive Substances**	**99**	**0,77**		**96,7**	**0,70**
1. Welcome the person and family (when present), in a private environment.	1,000	-	1. Welcome the person and family (when present), in a private environment.	1,000	-
2. Provide listening, identify main complaint, characteristics of suffering, life history, clinical conditions, context of use and build bonds.	1,000	-	2. Provide listening, identify main complaint, characteristics of suffering, life history, clinical conditions, context of use and build bonds.	1,000	-
3. Offer access to health care and guidance to prevent harm related to use and build bonds.	1,000	-	03. Offer access to health care and guidance to reduce harm related to use during listening, seeking to build bonds.	1,000	-

4. Carry out evaluation regarding consumption using tracking instruments[a,b]	1,000	0,650	04. Use the AUDIT questionnaire to investigate the quantity, frequency, abstinence symptoms associated with alcohol and associated problems.	0,937	0,557
5. Adopt brief intervention strategies [c]	1,000	0,702	5. Adopt brief intervention strategies[c.]	1,000	0,702
6. Suggest attendance at CECCO [d] or other spaces in the community network.	1,000	0,609	6. Suggest attendance at CECCO [d] or other spaces in the community network	1,000	0,609
7. Consider that if the individual is not in serious psychic distress, maintaining a functional relation with the different fields of life, they should maintain care in the PHC unit and/or street clinic.	0,938	0,582	7. Consider that if the individual is not in serious psychic distress, maintaining a functional relation with the different fields of life, they should maintain care in the PHC unit and/or street clinic.	0,938	0,582
8.Consider that, if it is a serious case, with intense suffering and harmful use, adding situations of clinical and social vulnerability, refer to PCC	1,000	0,621	08. Consider that, if it is a serious case, with intense suffering in which the person makes harmful use of drugs, adding situations of clinical and social vulnerability, share the care with PCC AD.	0,875	0,521

9. Consider referral to the general hospital and connecting EMCS in cases of acute poisoning with clinical repercussions.	1,000	0,609	9. Consider referral to the general hospital and connecting EMCS in cases of acute poisoning with clinical repercussions.	1,000	0,609
10. Consider referral to the general hospital, connecting EMCS in cases of harmful use with agitation and/or self- or hetero-directed aggression, refractory to the approach.	1,000	0,609	10. Consider referral to the general hospital, connecting EMCS in cases of harmful use with agitation and/or self- or hetero-directed aggression, refractory to the approach.	1,000	0,609
11. Consider referring the person to the general hospital, upon contacting EMCS in cases of consumption of alcohol and other drugs, which culminate in self-inflicted violence or suicidal ideation with structured planning and/or a consolidated suicide attempt in a recent episode.	1,000	0,582	11. Consider referral to the general hospital, connecting EMCS in cases that culminate in self-inflicted violence or suicidal ideation with structured planning and/or a consolidated suicide attempt in a recent episode.	1,000	0,582
12. Consider referring the individual to the general hospital, by connecting the EMCS , in cases of consumption of alcohol and other drugs that culminated in an attempt to harm themselves or leave the space to carry out the act.	1,000	0,609	12. Consider referral to the general hospital, connecting EMCS in cases that culminated in an attempt to injure oneself or leave the space to carry out the act.	1,000	0,609

13. Consider referral to the general hospital, connecting EMCS in cases of delirium or abstinence.	1,000	0,588	13. Consider referral to the general hospital, connecting EMCS in cases of delirium or abstinence.	1,000	0,588
14. Carry out follow-up after any emergency service/hospital discharge resulting from mental health hospitalization.	1,000	-	14. Carry out follow-up after any emergency service/hospital discharge resulting from mental health hospitalization.	1,000	-
			15. Use the CAGE[b] questionnaire to detect harmful use of alcohol.	0,937	0,546
			16. Use the ASSIST [e] questionnaire to check whether the person has used drugs in the last three months and evaluate dependence.	0,937	0,546
			17. Carry out brief intervention in the harmful use of psychoactive substances, using FRAMES [f]	0,937	0,557
			18. Suggest, in cases of addiction, the individual's attendance at self-help groups, such as Alcoholics	0,875	0,717

			Anonymous (AA) and Narcotics Anonymous (NA).		
**F- Nursing actions towards people in psychological distress as a result of grief situations**	**98**	**0,28**		**92**	**0,93**
1. Keep the listening attentive and empathetic in order to understand what is happening and letting her talk about the loss.	1,000	-	1. Keep the listening attentive and empathetic in order to understand what is happening and letting her talk about the loss.	1,000	-
2. Advise that mourning is a normal process in the face of an important loss.	1,000	0,099	2. Advise that mourning is a normal process in the face of an important loss.	1,000	0,099
3. Advise that grief has mental and physical effects.	1,000	0,008	3. Advise that grief has mental and physical effects.	1,000	0,008
4. Advise that people experience grief in different ways, some show strong emotions while others do not, and that crying does not mean weakness.	1,000	-	4. Advise that people experience grief in different ways, some show strong emotions while others do not, and that crying does not mean weakness.	1,000	-

5. Explain that, in most cases, grief lessens over time, and feelings of sadness, longing and pain will never disappear, but in most cases they ease over time.	0,938	0,051	5. Explain that, in most cases, grief lessens over time, and feelings of sadness, longing and pain will never disappear, but in most cases they ease over time.	0,938	0,051
6. Explain that sometimes it may be possible to feel good for a while and then something reminds of the loss and the individual feels as bad as them did in the beginning.	1,000	-, 143a	6. Explain that sometimes it may be possible to feel good for a while and then something reminds of the loss and the individual feels as bad as them did in the beginning.	1,000	-, 143a
7.Explain that there is no right or wrong way to experience grief and that sometimes it is possible to feel very sad; at other times, numb; until the individual can have fun, but, in general, these experiences become less intense as time passes.	1,000	-	7. Explain that there is no right or wrong way to experience grief and that sometimes it is possible to feel very sad; at other times, numb; until the individual can have fun, but, in general, these experiences become less intense as time passes.	1,000	-
8. Discuss the possible implementation of adaptation processes, or culturally appropriate ones, in the case of the loss of a loved one.	1,000	-, 208a	8. Discuss the possible implementation of adaptation processes, or culturally appropriate ones, in the case of the loss of a loved one.	1,000	-, 208a

9. Evaluate prolonged grief and discuss the case as a team for possible referral to PCC.	0,936	0,370	9. Evaluate prolonged grief and discuss the case as a team for possible referral to PCC	0,936	0,370
			10. Show empathy about the individual's grief.	1,000	-
			11. Encourage the person to express feelings about the loss.	1,000	0,918
			12. Help the person identify personal coping strategies.	1,000	0,918
			13. Help the person by bringing reality data to what happened.	1,000	0,918
			14. Reflect with the person that mourning is a natural process in the face of an important loss.	1,000	-
			15. Recognize as normal characteristics of grief in an initial period: somatic or physical stress, concern about the image of the person lost, guilt regarding the	1,000	-

	person or circumstance of their death, hostile reactions of nonconformity.		
	16. Recognize signs that the grief is not evolving properly: such as: the person cannot speak without presenting intense and recent feelings, minor events trigger an intense or disproportionate grief reaction.	1,000	0,927
	17. Recognize other signs that grief is not progressing properly, including: the person does not want to touch the “deceased” person's belongings, presents physical symptoms similar to those of the person who died and radical changes in lifestyle, subclinical depression and/or mania.	1,000	0,906
	18. Recognize other signs that grief is not evolving properly, including: compulsion to imitate the lost person, self-destructive impulses, seasonal depression, phobia of	1,000	0,918

	illness and death, stationary or decreasing performance.		
	19. Mobilize support networks in the community that can support the individual's grieving process.	1,000	-
	20. Advise the individual that grief has mental and physical effects, such as emptiness, tightness in the chest, lump in the throat, shortness of breath, lack of energy, muscle weakness, disbelief, sleep and appetite disorders, social isolation, dreams of the person who passed away, crying, visiting places, carrying significant objects.	1,000	0,947
	21. Evaluate the person regarding prolonged grief disorder and, in this case, discuss the case in a multidisciplinary team (e-Multi) and, if necessary, possible sharing of care with the PCC.	1,000	0,906

Source: Author.

IVC -Content Validation Index.

[1]Cronbach's alpha if item is deleted.

[a] AUDIT - for identifying problems with alcohol use;

[b] CAGE- in more serious cases to evaluate alcohol use;

[c] Suggest that the person keep a diary about their substance use, recording where they use it, how much they use, in which company they use it, reason for;

[d] Coexistence and Cooperative Center;

[e]ASSIST - Alcohol, Smoking and Substance Involvement Screening Test;

[f]FRAMES - F- Feedback, R- Responsibility, A- Advice, M- Menu of Options, E- Empathy, S- Self- efficacy
