# Supplementary material for: Construction and Validation of Nursing Actions to Integrate Mobile Care–Educational Technology to Assist Individual in Psychic Distress
Source: Int J Environ Res Public Health. 2025 Mar 13;22(3):419. doi: 10.3390/ijerph22030419 (PMC11941836; doi:10.3390/ijerph22030419)
Supplement: Supplementary file 1 [file ijerph-22-00419-s001.zip › Additional files- Questionnare 1.pdf]

QUESTIONNAIRE S1

TERM OF FREE AND INFORMED CONSENT (TCLE) NURSES

Dear Sir or Madam,

I invite you to take part in the research entitled "Construction and content validation of an educational technology for nursing care for adult users in psychological distress in primary health care" developed by Mr. Dárcio Tadeu Mendes. The aim of the research is to build and validate the content of a care-educational technology, such as a booklet, which can support Primary Health Care nurses in providing care to adult users in psychological distress.

If you accept, you will take part in individual interviews lasting an average of 40 minutes. The interviews will take place within the basic health unit and we will fill in an instrument, constructed by the researcher, with closed questions on identification data and sociodemographic profile and guiding questions that will aim to assess your understanding of psychological distress and your experience in caring for people in psychological distress. The results of this research could help Primary Health Care nurses to provide care to adults in psychological distress.

We present this Informed Consent Form so that you can assess the possibility of your participation in the research. Your answers are confidential and there is no need to disclose your personal details such as name and telephone number. We will be rigorous and responsible in analyzing the data you provide, as a way of offering correct information to the population. We guarantee the confidentiality of the data provided, and that only the researchers responsible will have access to it, as well as that all the information will be used exclusively for the purposes of this study.

You may experience discomfort such as physical/mental fatigue due to the time

taken to answer the questions. If you report that you are uncomfortable with the way the research is being conducted and/or while answering the questions, we will terminate your participation in the data collection and provide you with an individual welcome in a separate environment to help you at this time. You are free to refuse to participate or to stop participating at any time without any penalty.

As for the benefits of your participation in the research and in the future, it should be noted that the construction of a care-educational technology - a booklet - will be able to subsidize Primary Health Care nurses in their assistance to users in psychological distress. You will not incur any financial costs or expenses as a result of your participation. If you incur any expenses as a result of your participation, please contact the researcher responsible to be reimbursed. There is the possibility of compensation if you suffer any damage as a result of the research.

At any stage of the study, you will have access to the researcher responsible for the investigation to clarify any doubts. The principal investigator is Mr. Dárcio Tadeu Mendes, who can be reached at Rua Vinte e quatro de Fevereiro, 49. Vila Nova. Telephone: (11) 971865069 e-mail: dtmendes@usp.br. If you have any concerns or questions about the ethics of the research, please contact the Research Ethics Committee (CEP) - Address - Av. Dr. Enéas de Carvalho Aguiar, 419 - Cerqueira Cesar - São Paulo/SP CEP - 05403-000 Telephone- (11) 30618858 e-mail - cepee@usp.br. This research meets all the specifications of Resolution 466 of December 12, 2012, which approves the guidelines and regulatory standards for research involving human beings.

Researcher's signature

Signature of Participant/Legal Representative

Date:___/_/___

IDENTIFICATION DATA, SOCIODEMOGRAPHIC PROFILE AND SCRIPT OF THEMATIC QUESTIONS TO BE ADDRESSED DURING THE INTERVIEWS

I. IDENTIFICATION DATA

Initials of name:______________Age (completed years):___________

II. SOCIODEMOGRAPHIC PROFILE

1. Sex:(1) Male (2) Female

2. Time since graduation (in years):________________________

2. Do you have a postgraduate degree?______If yes, in which area? _________

4. Do you have any specific training in mental health? ( ) yes ( ) no.

If so, which one? __________________

5. Time working in Primary Health Care (PHC) -----

III. SCRIPT OF THEMATIC QUESTIONS

1. Tell me about your experience of caring for a user in psychological distress (Triggering question).

2. What do you think suffering is?

3. And what is your understanding of psychological suffering?

4. When you're dealing with a user, how do you identify that he or she

(a) is in psychological distress?

5. What do you do to intervene when you come across a user in psychological distress?

6. Do you use any specific instrument to assess and identify the user who is in psychological distress? If so, which one(s)?

7. When a user in psychological distress needs care from other services in the network, how do you share care?

8. Tell me about the circumstance(s) in which you refer the patient to another service.

(a) user in psychological distress.

9. When you refer to the user in psychological distress, there are defined criteria. If so, which ones?

10. Can you tell us whether or not there are any group activities here in the unit that are aimed at the participation of users in psychological distress? If so, under what conditions are they invited to take part?

11. If you were to add an instrument that could support your assessment when you come across a user in psychological distress, what would you add?

TERM OF FREE AND INFORMED CONSENT (TCLE) EXPERT JUDGES

Dear professional, I invite you to take part in the research entitled "Construction and validation of the content of an educational technology for nursing care for adult users in psychological distress in primary health care", developed by Mr. Dárcio Tadeu Mendes, whose objective is to construct and validate the content of a care-educational technology, such as a booklet, which can support Primary Health Care nurses in providing care for adult users in psychological distress.

The results of this research could help Primary Health Care nurses to provide care to adult users in psychological distress. If you accept, your participation implies answering an electronic questionnaire in Google Forms format sent by e-mail, with questions related to your profile as an interviewee and objective questions (closed questions) and subjective questions (open questions), referring to the content of the booklet in terms of its objectives, structure/presentation and relevance. It will take you an average of 40 minutes to respond.

Your participation will not bring you direct personal benefit, but your notes will be important in helping to build an educational technology, since you have extensive experience in the area of mental health research and work. Your answers are confidential and it is not necessary to disclose your personal details (such as name, telephone number), only an e-mail address will be required. All rigor and responsibility will be maintained in the quality of the data analysis, as a way of providing reliable information to the population.

Only the researcher will have access to the data provided. We guarantee the confidentiality of the data provided. We would like to inform you that the risks of taking part lie in the discomfort of reading the contents of the booklet and being asked questions

in the evaluation tool in a virtual environment. These will cease as soon as participation is concluded or if you decide not to continue with the research.

As for the benefits of your participation in the research and in the future, we would like to highlight your important contribution to the construction of a care-educational technology - a booklet that can support Primary Health Care nurses in their assistance to users in psychological distress.

You are free to refuse to take part or to stop taking part in the research at any time without any penalty. The results of this research will be presented at congresses in the field and published in a scientific journal, always guaranteeing the confidentiality of the names of the participants.

No financial costs are expected as a result of your participation in the study, but if you incur any costs as a result of your participation, we ask that you contact the researcher responsible in order to be compensated. There is also the possibility of compensation for any damage resulting from the research.

At any stage of the study, you will have access to the researcher responsible for the investigation to clarify any doubts. The principal investigator is Mr. Dárcio Tadeu Mendes, who can be reached at Rua Vinte e quatro de Fevereiro, 49. Vila Nova. Telephone: (11) 971865069 e-mail: dtmendes@usp.br.

If you have any concerns or questions about the ethics of the research, please contact the Research Ethics Committee (CEP) - Address - Av. Dr. Enéas de Carvalho Aguiar, 419 - Cerqueira Cesar - São Paulo/SP CEP - 05403-000 Telephone- (11) 30618858 e-mail - cepee@usp.br

This research meets all the specifications of Resolution 466 of December 12, 2012, which approves the guidelines and regulatory standards for research involving human beings.

Researcher's signature

.

Signature of Participant/Legal Representative

Date:___/_/___

JUDGES-EXPERTS QUESTIONNAIRE: FIRST ROUND

Dear specialist, the purpose of this research is to validate Nursing Actions in order to build the content of a care-educational technology that can support Primary Health Care nurses in providing care to adult users in psychological distress. In accordance with the invitation previously accepted, below is the material for the validation study.

In this questionnaire, you are asked to give your technical opinion on the most appropriate **Nursing Actions** to be taken by nurses working in Primary Health Care (PHC) when faced with the demands of people in psychological distress, in the following presentations: Approach to the person in common mental suffering; Approach to the person with complaints associated with depressive disorder; Approach to the person with complaints associated with anxiety disorder; Approach to the person with suicidal ideation; Approach to the person in psychological suffering due to the use of psychoactive substances and Approach to people exposed to bereavement situations.

The questionnaire consists of two parts:

Part I: **Expert data -** Characterization of the panel of experts, with questions relating to age, gender, training and professional experience, area of performance and experience/work carried out in the area of mental health/intervention with people suffering from mental illness; Part II: **Nursing actions in the various presentations of psychological distress -** A consideration of psychological distress is presented, followed by the nursing actions to be

evaluated (in the various contexts of presentation of psychological distress).

Instructions for filling in the form:

- Please indicate your level of agreement* with each **Nursing Action** presented;

* The level of agreement referred to by the expert judges in the instrument sent

was indicated using a Likert scale: totally agree, partially agree, no opinion, partially disagree and totally disagree.

At the end of each presentation of psychological suffering, with its respective **Nursing Actions,** there is a space for comments and/or suggestions and the possibility of additions, if you think it is pertinent to this research; We thank you for your valuable cooperation and ask that you complete and return the questionnaire within **15 days**.

PART I - EXPERT DATA

Please fill in the information requested.

1. Sex ( ) Female ( ) Male
2. Age:__years
3. Time as a graduate?
4. Specialization/Residency in: ( ) Mental Health and Psychiatry ( ) Collective Health ( ) Public Health ( )
5. Master's degree in: ( ) Mental and Psychiatric Health, ( ) Collective Health, ( ) Public Health ( ) No Master's degree
6. Doctorate in: ( ) Mental and Psychiatric Health ( ) Collective Health ( ) Public Health ( ) No Doctorate
7. Post-doctorate: Yes ( ) No ( )
8. Time (in years) of professional experience (clinical and care) in the area of training as a Specialist:____
9. Time (in years) of professional activity (clinical-assistance) area of training as Master:_______
10. Time (in years) of professional experience (clinical and care) in the area of training as a Doctor:_

11. Time (in years) of professional activity (teaching) in the area of training as a Specialist:___

12. Time (years) of professional activity (teaching and research) in the area of training as Master:____

13. Time (years) of professional activity (teaching and research) in the area of training as a Doctor:________________________

14. Are you a member of a Scientific Society/Department/Chapter in the following thematic area: ( ) Mental and Psychiatric Health ( ) Collective Health ( ) Public Health ( )

15. Experience with product construction and validation studies (scales, games, apps) in the subject area: ( ) Yes ( ) No

PART II- NURSINGACTIONS IN THE VARIOUS PRESENTATIONS OF PSYCHOLOGICAL DISTRESS PSYCHOLOGICAL DISTRESS (CONTEXTUALIZATION)

This research adopted Cassell's Theoretical Framework (Cassell, 2004) from Caderno 34 of the Ministry of Health (Brazil, 2013), which defines psychological suffering as "the experience of the threat of rupture of the person's unity/identity and can be described as common mental suffering or psychoses".

As a historical and social being, a person's psychological distress is related to the challenges they commonly face in their daily lives, including structural factors such as gender, skin color, income, schooling and work, and situational factors such as marital conflicts, conflicts at work and the loss of a loved one, which also increase the risk of psychological distress. It is also the result of the emotional impact on the person's life, their temperament, their life history and their support network. Therefore, very often, people report that some significant event in their lives preceded the onset of psychological distress.

Adding Goldberg & Huxley's systematized concept of Common Mental Disorder (CMD) to designate nonspecific somatic complaints without the presentation of psychotic signs and symptoms, which do not meet the criteria for depressive, anxiety and/or somatoform disorders, according to ICD 10 and DSM V, but which produce functional incapacity comparable to and sometimes greater than chronic mental disorders, representing one of the most important causes of morbidity in Primary Health Care (PHC).

According to the World Health Organization (WHO), few cases are diagnosed in PHC and they are often underestimated, especially when physical symptoms are not

present, resulting in inadequate treatment and users making pilgrimages to health services. The WHO points out the importance of PHC in developing actions to promote mental health, with screening, referral and monitoring of people with mental disorders.

In the context of PHC, people seek help with non-specific complaints, which are often not immediately recognized by health professionals due to the greater value placed on physical complaints.

It is in PHC that people with CMD seek to be welcomed, understood and attended to in their needs, and they are attended to by nurses, who need to have mastery of technical and scientific knowledge in order to offer resolutive mental health actions and take co-responsibility for the person as a whole.

In this scenario, the medical diagnosis is made on the basis of manifestations of mild to moderate intensity according to two main diagnostic categories, according to the concept of CMD: Depressive Disorders - Major Depression, Depressive Episode and Dysthymia and; Anxiety Disorders - Generalized Anxiety Disorder (GAD), Obsessive Compulsive Disorder (OCD), Post-Traumatic Stress Disorder (PTSD), Panic Disorder, phobias, Social Anxiety Disorder.

2-ACTIONS TOWARDS PEOPLE IN PSYCHOLOGICAL DISTRESS (CONTEXTUALIZATION)

The construction of the Nursing Actions to be evaluated followed the following steps:

1-Scope Review (actions used by PHC nurses to support adult users in psychological distress); Qualitative research with nurses who work in PHC (to understand how they conduct nursing actions towards people in psychological distress in PHC); Adoption of national and international *guidelines* on how to conduct actions towards people in psychological distress in PHC): adoption of national and international *guidelines* on how to conduct actions towards people in psychological distress in PHC): Primary Care Notebook, Nº34 (Brazil, 2013);

2- World Organization of Family Doctors (WONCA, 2017);

3. Manual of Interventions for mental, neurological and alcohol and other drug use disorders in the Primary Health Care network (MI-mhgap) (2018);

4. Addressing common mental suffering in primary care in 7 steps (Castro and Wenceslau, 2023);

5. Babel Card for Mental Health in Primary Care (Rio de Janeiro: CEPESC, 2009);

6. Lines of Care Primary Care Secretariat. Depression in adults (Ministry of Health, 2022);

7. Referral Protocols for Adult Psychiatry (Telessaúde RS- UFRGS, 2016);

8. Lines of Care Primary Care Secretariat. Anxiety disorders in adults (Ministry of Health, 2022);

9. Prevention of suicide risk: guide for health professionals. Ponta Grossa - PR: Atena (Martin, Silva, Pedrollo et.al, 2022).

10. Quick Reference Guide Collection, Suicide Risk Assessment and Prevention. Professional Version (Rio de Janeiro. City Hall, 2016);

11. Strategic guide for the care of people with needs related to the consumption of alcohol and other drugs (Ministry of Health. 2015);

12. Quick Reference Guide Collection, Alcohol and other drugs (Rio de Janeiro. City Hall, 2016);

13. Guidelines on mental health crisis care and the longitudinal monitoring of cases in the Psychosocial Care Network in the Municipality of São Paulo. Mental Health Risk Stratification and Classification (City of São Paulo, 2023);

14. Primary Care Nursing Protocol. Fascículo A Enfermagem no Cuidado em Saúde Mental no Contexto da Atenção Primária à Saúde (Rio de Janeiro. City Hall, 2017);

15. Classification of Nursing Interventions (NIC)- 7.ed. (2022);

16. Mental health in primary care: multiprofessional approach -1. ed. (Fernandes, 2022);

17. Suicide crisis: assessment and management - 2. ed. (Botega, 2023), 18. Use of Assessment Scales: Nurses Global Assessment Risk Suicide 1, AUDIT 2, CAGE 3 and ASSIST 4.

Scale designed in the United Kingdom and recently validated for the Brazilian population, intended for use by professional nurses during nursing consultations, consisting of 15 simple scoring items, which include predictive variables for suicidal behavior, allowing classification into four levels of suicide risk, which should serve as a parameter for decision-making about the necessary intervention: low risk (score less than or equal to five); risk intermediate (score between 6 and 8), high risk (score between 9 and 11) and very high risk (score greater than or equal to 12) (Veloso, Monteiro, Santos, 2021).

The AUDIT (Alcohol Use Disorders Identification Test) instrument was developed by the World Health Organization (WHO). Composed of 10 questions, it aims to identify possible alcohol dependence (Méndez et al., 1999).

The CAGE questionnaire - an acronym referring to its four questions - Cut down, Annoyed by criticism, Guilty and Eye-opener. It is used with a cut-off point of two affirmative answers suggesting positive *screening* for alcohol abuse or dependence (Masur, Monteiro, 1993).

Alcohol, Smoking and Substance Involvement Screening Test. It is a brief screening questionnaire to detect people who use psychoactive substances. It was developed by the WHO by researchers from various countries to detect the use of alcohol, tobacco and other drugs of abuse. It is indicated for use in PHC (Henrique et al., 2004). Based on this information, please look at each item and, on a Likert scale, give your opinion:

**NURSING ACTIONS IN THE INITIAL ASSESSMENT OF PEOPLE IN PSYCHOLOGICAL DISTRESS**
1. active listening, allowing the person to speak without being interrupted.
2. Make sure that the person does not have a serious mental disorder that will require another approach, paying attention to warning conditions: suicidal ideation and risk of self/hetero-aggression, manic episode, altered level of consciousness, presence of psychotic symptoms, association with serious or decompensated physical illness: Diabetes, Hypothyroidism, Lupus, Parkinson's disease, nutritional deficiency.

3. Explore, during active listening, the person's psychosocial status and experience of illness, using the BATHE and SIFE techniques, respectively.<br>BATHE TECHNIQUE<br>B-Background - Ask the person how life has been? What has been happening? A-Affect - Ask the person how they feel about the situation.<br>T-Troubles - Ask the person what kind of problems and difficulties this situation brings. H- Handling - Asking the person how they have dealt with the situation.<br>E-Empathy: "I realize that you're facing a (very) difficult time. SIFE TECHNICAL<br>S- Feelings - the person's feelings about their problem/suffering - For example, ask: How do you feel about (problem/suffering)? I- Ideas- the person's ideas about what is wrong. Ask, for example: What do you think might be causing this problem/suffering?
F- Functioning - effects of the illness on people's functioning - Ask, for example: How does this problem/suffering influence the things you do on a daily basis?<br>E- People's expectations of the care to be offered - How do you think I could help you?
4. Demonstrate an empathetic response to the person's problem while listening, using the NURSE technique.<br>NURSE TECHNICIAN<br>N- Naming - "It seems to me that you are concerned about these issues". U-Understanding - "I understand how much this has been worrying you." R- Respecting - "It must be very distressing to deal with (...)".<br>S- Supporting "I'd like to know how you expect me to help you". E- Exploring. How are you coping?
5. carry out a physical examination of the person and measure blood pressure, respiratory rate and heart and lung auscultation.

6. Assess whether there is a physical cause that explains the symptoms reported by the person and, if there is, verify strategies for conducting treatment.
7. Understand and explain what really led the person to go to the unit. (Clarifications - For example, ask: "What does it mean to you to feel unwell?<br>/ Recaps - Offer the person a summary of the information and conclude with questions. For example: Do you think this summary reflects what happens to you?)
8. Contextualize suffering in a person's life, in its various dimensions, from the individual to the social and collective.<br>For example, ask: "Have you ever felt this way at other times in your life? How did you deal with it?"
9. Removing the problem that the person presents as something pathological that needs to be medicalized.<br>For example: Reflect with the person that all the suffering they are experiencing can, unfortunately, happen to many people who are going through difficult situations like theirs, and that although I understand the situation, it is possible that they will feel this way for some time to come.
10. Carry out psychoeducation with the person in psychological distress. (For example: Tell the person that, in view of what you have discussed, you understand that they have been experiencing suffering/illness, sometimes with physical manifestations that are deeply related to the difficult situations they are facing).
11. Develop the person's problem-solving skills, conveying the message that they are healthy, despite their expected suffering, and that they have the tools to deal with this problem (For example: I want to reinforce that you don't have a disease and that medication

won't help you solve your problems; that mental health care doesn't always involve giving diagnoses and prescribing medication).<br>Problem-solving therapy (depending on the time available and the person's wishes), helping the person to identify and solve their own problems in a structured and sequential way. It's a task that can be done by the person at home and presented at a future appointment. The person should be asked to: Build an objective list of the main problems they presented during the listening session and consider their complexity, priority and urgency; Brainstorm possible solutions to the problem presented; Choose the solution they believe is most likely to succeed and that they are willing to implement; Choose a day and time to apply their plan; Think about situations that could interfere with success and develop a coping plan.
12. Developing a bond with the person and securing their trust. (Reinforcement - For example: I think today's consultation was important for us to better understand your suffering and the issues related to it. / Verification - For example: I'd like to know if you have any questions about what we talked about today. / Agreeing on a follow-up - For example: I thought I'd arrange to come back in two weeks to see how things are going. / Closing - Eye contact and a farewell greeting).
13. Comments/Suggestions. If "yes", please specify. If not, adopt "no".
14. Would you like to add any nursing actions? If "yes", please specify. If not, choose "no".

NURSING ACTIONS FOR PEOPLE IN PSYCHOLOGICAL DISTRESS WITH COMPLAINTS ASSOCIATED WITH DEPRESSIVE DISORDER
1.Identify whether, in the last two weeks, you have felt sad, discouraged, depressed, for most of the day, almost every day.
2. Identify whether in the last two weeks the person has felt that they no longer enjoy anything, that they have lost interest and pleasure in the things they used to enjoy.
3. Make sure that if there is at least one "yes" answer in items 1 or 2, the questions in actions 4 to 10 must be asked.
4. Ask the person if their appetite has changed significantly.
5. Check if the person has had sleep problems almost every night (difficulty falling asleep, waking up in the middle of the night, sleeping too much).
6. Check whether the person has been moving more slowly than usual or, on the contrary, has felt more agitated or unable to sit still.
7. Check if the person feels tired most of the time, without energy, almost every day.
8. Check if the person feels worthless or guilty almost every day.
9. Check if the person has difficulty making decisions, concentrating or memory problems almost every day.
10. Check whether the person has had bad thoughts several times, such as: "It would be better to be dead" or to harm themselves.
11. Consider that if the person's answer was "yes" to actions 1 or 2 and "yes" to any of the questions in actions 4 to 10, there is a high risk of depression.
12. Consider that if there are 3-4 positive answers, it is a mild depression.
13. Consider that if there were 05 to 07 positive responses, it is moderate depression.
14. Consider that if there were 08 to 09 positive answers, it is a case of severe

depression.
15. Consider that a person with depression may also present symptoms of anxiety and clinically unexplained somatic symptoms.
16. Assess whether the person is at risk of suicide.
17. Check whether the person has Bipolar Affective Disorder, investigating previous mania.
18. Question the person about their use of alcohol and drugs.
19. Offer psychoeducation to the person and their family/caregivers: during the assessment or through groups on what depression is, its symptoms, treatment and in cases of thoughts of self-harm/suicide, the person should tell someone they trust and go to the PHC unit.
20. Guide the person to resume or continue with activities that were previously pleasurable.
21. Guide the person to regular bedtimes and wake-up times.
22. Encourage the person to try to keep eating regularly despite variations in appetite.
23. Reinforce to the person the importance of participating in daily activities and social/community life.
24. Consider that if the person does not improve after being re-evaluated in 6 to 8 weeks, the case should be discussed with the Extended Family Health Center (NASF) team with a view to being referred to the Psychosocial Care Center (CAPS).
25. Consider that if the person is in a possible depressive phase of bipolar affective disorder, they should undergo a medical evaluation with a professional from the primary care unit and be considered for referral to the Psychosocial Care Center (CAPS) after discussing the case with a team from the Expanded Family Health Center (NASF).
26. Consider referring the person to the Psychosocial Care Center (CAPS), through a

referral and counter-referral guide, in cases of severe depression with psychotic symptoms or catatonia.
27. Consider referring the person to the Psychosocial Care Centre (CAPS), using a referral and counter-referral guide, in cases of previous severe depressive episodes - psychotic symptoms, suicide attempts or psychiatric hospitalization.
28. Consider referring the person to the Psychosocial Care Center (CAPS), using a referral and counter-referral guide, in cases of depression and persistent suicidal ideation after initial management in Primary Health Care (PHC) (no improvement 12 weeks after the start of management).
29. Consider referring the person to the emergency service for immediate assessment, using a referral and counter-referral form and calling the Mobile Emergency Care Service (SAMU), if psychotic symptoms appear (ideas of guilt, misery, talking to oneself, wandering).
30. Consider referring the person to the emergency service for immediate assessment, using a referral and counter-referral form and calling the Mobile Emergency Assistance Service (SAMU), if there is an association of acute suicidality (current suicidal ideas with a concrete plan, access to lethal means, visible despair and hopelessness).
31. Consider referring the person to the emergency department for immediate assessment, using a referral and counter-referral form and calling the Mobile Emergency Care Service (SAMU), if there is an association of very marked neurovegetative signs and symptoms: delayed verbal response, motor slowing, weight loss due to inappetence.
32. Consider referral to the emergency service, using a referral and counter-referral form and calling the Mobile Emergency Care Service (SAMU) in cases where there are clinical, surgical, obstetric or psychiatric complications (intoxication or withdrawal from substances), agitation, aggression and/or impulsivity.
33. Follow up on the person after they have been treated in an emergency service/are

discharged from hospital as a result of being admitted to a mental health ward.
34. Comments/Suggestions. If "yes", please specify. If not, adopt "no".
35. Would you like to add any nursing actions? If "yes", please specify. If not, choose "no".

NURSING ACTIONS FOR PEOPLE IN PSYCHOLOGICAL DISTRESS WITH COMPLAINTS ASSOCIATED WITH ANXIETY DISORDER
1. Check if the person has been worrying too much.
2. Check if the person has been feeling exhausted or tense.
3. Check if the person has been feeling very irritable or has a "nervous breakdown"?
4. Check if the person has difficulty relaxing.
5. Consider that if the person's answer was "yes" to at least two of the actions covered by items 1 to 4, the questions covered by actions 6 to 10 should be asked.
6. Check if the person has been sleeping badly or has difficulty sleeping.
7. Check whether the person has had a headache, neckache or headache.
8. Check for dizziness, cold sweats, diarrhea, tingling, stomach discomfort, churning.
9. Check if the person is worried about their health.
10. Check whether the problems reported by the person have affected their quality of life and relationships with other people.
11. Consider that if there have been 05 or more positive responses over at least 06 months, this indicates a strong risk of the person having a diagnosis of an anxiety disorder.
12. Offer psychoeducation to all people, their families and caregivers during the assessment or through information groups about what anxiety is and the techniques that can reduce symptoms.
13. During individual or group psychoeducation, instruct people that in cases of anxiety attacks, they can use diaphragmatic breathing: place one hand on their stomach and the other on their chest and only the hand on their stomach should move while they breathe slowly through their nose.

14. Instruct the person during individual or group psychoeducation to develop processes of reflection on the rationale of thoughts in cases where negative or unpleasant thoughts arise.
15. Instruct the person during individual or group psychoeducation not to try to push negative thoughts away, emphasizing that the important thing is not to attach importance to them and that they will disappear over time.
16. Offer people the chance to take part in Integrative and Complementary Practices groups.
17. Reassure the person in panic attacks (sudden and unexpected sensation of terror, associated with autonomic symptoms, particularly cardiorespiratory symptoms), informing them that the symptoms are due to an anxiety attack, unrelated to a serious medical condition with an imminent risk of death.
18. Reassure the person that panic attacks are temporary (only a few minutes).
19. Instruct the person in panic attacks to breathe through their nose and not through their mouth, so as not to hyperventilate.
20. Request medical assessment in cases where the person has intense and prolonged crises.
21. Consider referring the person to the Alcohol and Other Drugs Psychosocial Care Center (CAPS AD), after discussing the case with the team or at matrix support meetings, in cases where there is an association with a serious psychoactive substance use disorder.
22. Consider referring the person to the CAPS, by discussing the case with the team or at matrix support meetings, in cases where there is a comorbidity with bipolar affective disorder or psychotic symptoms.
23. Consider referring people with anxiety disorders and persistent suicidal ideation

after initial management in Primary Health Care (PHC) to the Psychosocial Care Center (CAPS) 12 weeks after the start of management without improvement.
24. Consider referral to CAPS, through team case discussions or matrix support meetings, for refractory people - lack of response or partial response to two effective therapeutic strategies (psychotropic drugs and/or psychotherapy) for at least 8 weeks.
25. Consider referring the person to the emergency service for immediate assessment, using a referral and counter-referral guide and calling the SAMU, if the person manifests the association of acute suicidality (current suicidal ideas with a concrete plan, previous attempts, substance abuse, access to lethal means, visible despair and hopelessness).
26. Follow up on the person after they have been treated in an emergency service/are discharged from hospital as a result of being admitted to a mental health ward.
27. Comments/Suggestions. If "yes", please specify. If not, adopt "no".
28. Would you like to add any nursing actions? If "yes", please specify. If not, choose "no".

NURSING ACTIONS FOR PEOPLE IN PSYCHOLOGICAL DISTRESS WITH SUICIDAL IDEATION
1. Listen to the person in a private setting, without interruptions, leaving them free to explain what is happening.
2. Use a calm, accepting, non-judgmental approach and pay attention to non-verbal expressions.
3. seeking to establish a therapeutic relationship of trust, empathy, authenticity and respect.
4. Welcoming the person in a comprehensive, careful and individualized way, encouraging them to recognize and express their feelings, in accordance with their needs and anxieties, encouraging them to understand what is happening.
5. To assess the person's risk of suicide, use the Nurses Global Assessment Risk Suicide Scale.
6. Promoting safety (supervision and restricting access to means of self-injury) in the event of a serious risk of suicide.
7. Help the person to see their strengths (for example, reinforce that the choice to seek help was a good one), validate their feelings and help them regain control.
8. Strengthen and identify the support network, if possible, with the person's consent.
9. Demonstrate that you accept the person's desire not to feel pain and convey your desire to support them in finding healthy alternatives for dealing with pain.
10. Thinking together with the person about therapeutic paths and healthy ways of dealing with suffering.
11. Involve family, friends and others in the risk assessment and treatment of suicide-related behavior, when appropriate and with the person's knowledge and consent, as they can

ensure monitoring, guarantee safety, prevent access to means and encourage compliance with treatment recommendations.
12. Establishing a follow-up plan with the person so that care can be continued, such as recognizing warning signs; identifying internal coping strategies; identifying interpersonal support as a means of distraction from unpleasant thoughts or impulses; contacting significant people to help resolve the crisis; contacting health services that have a care link; reducing potential access to lethal means.
13. Consider that if there are severe depressive symptoms and plans to commit suicide, the person should be referred to the general hospital to be assessed for the need for hospitalization or to the Psychosocial Care Center (CAPS) for intensive monitoring and the case should be shared with another person. Team professional (doctor, psychologist from the Expanded Family Health Center (NASF), for example).
14. Call the Extended Family Health Center (NASF) / Psychosocial Care Center (CAPS) at the time of the visit to discuss the case, if there are any doubts during the person's visit.
15. Consider that, once the condition has stabilized, the person's care should be maintained in the PHC unit, in order to monitor suicidal ideation in relation to its progression to planning and attempting.
16. Comments/Suggestions. If "yes", please specify. If not, adopt "no".
17. Would you like to add any nursing actions? If "yes", please specify. If not, choose "no".

NURSING ACTIONS FOR PEOPLE IN PSYCHOLOGICAL DISTRESS DUE TO THE USE OF PSYCHOACTIVE SUBSTANCES
1. welcome the person and their relative (if present) in a private environment).
2. listening to the person and identifying the main complaint and characteristics of suffering, life history, clinical conditions, context of drug use, seeking to build a bond.
3 Offering the person access to health care and guidance to prevent harm related to use during the listening session, seeking to build a bond.
4. Carry out an assessment of the person's consumption of psychoactive substances, using screening instruments.<br>*AUDIT- for the identification of alcohol use problems;<br>**CAGE- in more serious cases to assess alcohol use;<br>***ASSIST- for cases of alcohol and other drugs.
5. Adopt brief intervention strategies for people who abuse alcohol and other drugs:<br>-Suggest that the person keep a diary of their substance use, recording where they use it, how much, in what company, why;<br>- Identify activities that give you pleasure and suggest doing them at times when you would be using the substance.
6. Suggest the city's Cultural and Social Center or other spaces in the community network.
7) Consider that if the person uses drugs, but is not in serious psychological distress and maintains a functional relationship with the various areas of life, they should be cared for at the PHC unit and/or street clinic.

8. consider that in the case of a severe case, with intense suffering and harmful use of drugs, adding to situations of clinical and social vulnerability, the person should be referred to CAPS AD.
9. consider referring the person to the general hospital by calling the SAMU in cases of acute intoxication by psychoactive substances (medication, alcohol and other drugs) with clinical repercussions and lowered level of consciousness, and/or psychomotor agitation and/or aggression involving risk to themselves or others.
10. Consider referring the person to a general hospital, by calling the SAMU, in cases of harmful use of alcohol and other drugs, with agitation and/or self- or hetero-directed aggression, refractory to treatment.
11. Consider referring the person to a general hospital, by calling the SAMU, in cases of alcohol and other drug use that culminate in self-inflicted violence or suicidal ideation with structured planning and/or a consolidated suicide attempt in a recent episode.
12. Consider referring the person to a general hospital, by calling the SAMU, in cases of alcohol and other drug use that result in an attempt to harm oneself or leave the space to carry out the act.
13. Consider referring the person to a general hospital by calling the SAMU in cases of harmful use of alcohol and other drugs, delirium or withdrawal.
14. Follow up on the person after they have been treated in an emergency service/been discharged from hospital as a result of being admitted to a mental health ward.
15. Comments/Suggestions. If "yes", please specify. If not, adopt "no".
16. Would you like to add any nursing actions? If "yes", please specify. If not, choose "no".

NURSING ACTIONS TOWARDS PEOPLE IN PSYCHOLOGICAL DISTRESS AS A RESULT OF BEREAVEMENT SITUATIONS
1) Maintain attentive and empathetic listening, in order to understand what is happening to the person and let them talk about the loss.
2. Educate the person that mourning is a normal process in the face of a major loss.
3. Advise the person that grief has mental and physical effects.
4. Point out that people experience grief in different ways, where some people show strong emotions while others don't, and that crying doesn't mean weakness.
5. Explain that, in most cases, grief diminishes over time, that you may think that the sadness, longing or pain will never go away, but in most cases, these feelings subside over time.
6. Explain that sometimes a person can feel fine for a while and then something reminds them of the loss and they feel just as bad as they did at the beginning.
7) Explain to the person that there is no right or wrong way to experience grief and that sometimes they will feel very sad, other times numb, and at other times they will even be able to enjoy themselves, but that, in general, these experiences will become less intense and less frequent as time goes by.
8. Approach the person about the possibility of carrying out adaptation or culturally appropriate processes in the event of the loss of a loved one.
9. Assess the person for prolonged bereavement disorder and, if so, discuss the case with the team for possible referral to CAPS.
10. Comments/Suggestions. If "yes", please specify. If not, adopt "no".
11. Would you like to add any nursing actions? If "yes", please specify. If not, choose "no".

APPENDIX E - JUDGES-EXPERTS QUESTIONNAIRE: SECOND ROUND

NURSING ACTIONS IN THE INITIAL ASSESSMENT OF PEOPLE IN PSYCHOLOGICAL DISTRESS
1. active listening, allowing the person to speak without being interrupted.
2. Make sure that the person does not have a serious mental disorder that will require another approach, paying attention to warning conditions: suicidal ideation and risk of self/hetero-aggression, manic episode, altered level of consciousness, presence of psychotic symptoms, association with serious or decompensated physical illness: Diabetes, Hypothyroidism, Lupus, Parkinson's disease, nutritional deficiency.
3. Explore, during active listening, the person's psychosocial status and experience of illness, using the BATHE and SIFE techniques, respectively.<br>BATHE TECHNIQUE<br>B-Background - Ask the person how life has been? What has been happening? A-Affect - Ask the person how they feel about the situation.<br>T-Troubles - Ask the person what kind of problems and difficulties this situation brings. H- Handling - Asking the person how they have dealt with the situation.<br>E-Empathy "I realize that you're facing a (very) difficult time. SIFE TECHNICAL<br>S- Feelings - the person's feelings about their problem/suffering - For example, ask: How do you feel about (problem/suffering)? I- Ideas- the person's ideas about what is wrong. Ask, for example: What do you think might be causing this problem/suffering?<br>F- Functioning - effects of the illness on people's functioning - Ask, for example: How does this problem/suffering influence the things you do on a daily basis?<br>E- People's expectations of the care to be offered - How do you think I could help you?

4. Demonstrate an empathetic response to the person's problem while listening, using the NURSE technique.<br>NURSE TECHNICIAN<br>N- Naming - "It seems to me that you are concerned about these issues". U- Understanding - "I understand how much this has been worrying you." R- Respecting - "It must be very distressing to deal with (...)".
S- Supporting "I'd like to know how you expect me to help you". E- Exploring. How are you coping?
5. carry out a physical examination of the person and measure blood pressure, respiratory rate and heart and lung auscultation.
6. Assess whether there is a physical cause that explains the symptoms reported by the person and, if there is, verify strategies for conducting treatment.
7. Understand and explain what actually led the person to go to the unit. (Clarifications - For example, ask: "What does it mean to you to feel unwell?<br>/ Recaps - Offer the person a summary of the information and conclude with questions. For example: Do you think this summary reflects what happens to you?)
8. Contextualize suffering in a person's life, in its various dimensions, from the individual to the social and collective.<br>For example, ask: "Have you ever felt this way at other times in your life? How did you deal with it?"
9. Removing the problem that the person presents as something pathological that needs to be medicalized.<br>For example: Reflect with the person that all the suffering they are experiencing can, unfortunately, happen to many people who are going through difficult situations like theirs, and that although I understand the situation, it is possible that they will feel this way for some

time to come.
10. Carry out psychoeducation with the person in psychological distress. (For example: Tell the person that, in view of what you have discussed, you understand that they have been experiencing suffering/illness, sometimes with physical manifestations that are deeply related to the difficult situations they are facing).
11. Develop the person's problem-solving skills, conveying the message that they are healthy, despite their expected suffering, and that they have the tools to deal with this problem.<br>(For example: I want to reinforce that you don't have a disease and that medication won't help you solve your problems; that mental health care doesn't always involve giving diagnoses and prescribing medication).<br>Problem-solving therapy (depending on the time available and the person's wishes), helping the person to identify and solve their own problems in a structured and sequential way. It's a task that can be done by the person at home and presented at a future appointment. The person should be asked to: Build an objective list of the main problems they presented during the listening session and consider their complexity, priority and urgency; Brainstorm possible solutions to the problem presented; Choose the solution they believe is most likely to succeed and that they are willing to implement; Choose a day and time to apply their plan; Think about situations that could interfere with success and develop a coping plan.
12. Develop a bond with the person and ensure their trust. (Reinforcement - For example: I think today's consultation was important for us to better understand your suffering and the issues related to it. / Verification - For example: I'd like to know if you have any questions about what we talked about today. / Follow-up agreement - For example: I thought I'd schedule a return visit in two weeks to see how things are going. / Closing - Eye contact and a farewell greeting).
13. Carry out psychoeducation on psychological suffering with the person. For

example: Tell the person that, in light of what you've discussed, you understand that they have been experiencing widespread suffering/illness, sometimes with physical manifestations, deeply related to the difficult situations they are facing.
14. Building tools with the person to deal with psychological suffering: For example: Helping the person to identify and solve their own problems, in a structured and sequential way. This can be done by the person at home and presented at the next appointment. The person should be asked to: 1. build an objective list of the main problems they presented during the listening session and consider their complexity, priority and urgency 2. brainstorm possible solutions to the problem presented. 3. choose the solution that they believe is most likely to succeed and that they are willing to implement. 4. choose a day and time to implement your plan. 5. think about situations that could interfere with success and develop a coping plan.
15. Develop trust and a bond with the person. Summarization- Summarize the content of the interaction by saying, for example: "Today we talked about this problem" (describe the problem objectively). Validation- Carry out validation by asking the person to repeat what was said during the nursing consultation. Follow-up agreement - For example: I thought I'd schedule a return visit in two weeks to see how things are going. Closing - Eye contact and a farewell greeting.

NURSING ACTIONS FOR PEOPLE IN PSYCHOLOGICAL DISTRESS WITH COMPLAINTS ASSOCIATED WITH DEPRESSIVE DISORDER
01. Identify whether in the last two weeks the person has felt sad, discouraged, depressed, for most of the day, almost every day.
02. Identify whether, in the last two weeks, the person has had the feeling that they no longer enjoy anything, that they have lost interest and pleasure in the things they used to enjoy.
03.Make sure that if there is at least one "yes" answer in items 1 or 2, the questions contemplated between actions 4 and 10 must be carried out.
04. Ask the person if they have noticed a difference in their appetite over the last few days.
05. Check if the person has had sleep problems almost every night (difficulty falling asleep, waking up in the middle of the night, sleeping too much).
06. Check whether the person has been moving more slowly than usual, not wanting to carry out everyday tasks or, on the contrary, has felt more agitated or unable to sit still.
07. Check if the person felt tired most of the time, without energy, almost every day.
08. Check if the person feels worthless or guilty almost every day.
09. Check if the person has difficulty making decisions, concentrating or memory problems almost every day.
10. Check whether the person has had bad thoughts several times, such as: "It would be better to be dead" or "to do harm to oneself".
11. Consider that if the person's answer was "yes" to actions 1 or 2 and "yes" to any of the questions in actions 4 to 10, there is a high risk of depression.
12. Consider that if there were 03 to 04 positive responses, there is an association with mild depression.

13. Consider that if there were 05 to 07 positive responses, there is an association with moderate depression.
14. Consider that if there were 08 to 09 positive responses, there is an association with severe depression.
15. Consider that a person with depression may also present symptoms of anxiety and clinically unexplained somatic symptoms.
16. Assess whether the person is at risk of suicide.
17. Check whether the person has bipolar disorder, investigating previous episodes of mania (euphoria)/depression.
18. Question the person about their use of alcohol and other drugs.
19. Offer psychoeducation to the person and their family/caregivers: during the assessment or through groups on what depression is, its symptoms, treatment and in cases of thoughts of self-harm, non-suicidal self-injury and suicide, the person should tell someone they trust and go to the Primary Health Care unit.
20. Guide the person to resume or continue with activities that were previously pleasurable or identify new ones.
21. Guide the person to regular bedtimes and wake-up times.
22. Advise the person on the importance of maintaining a fractioned diet, preferring nutritious and caloric foods and reducing stimulating drinks such as coffee, mate tea and alcohol.
23. Reinforce to the person the importance of participating in daily activities and social/community life.
24. Consider that if there is no improvement in the person after the reassessment in 6 to 8 weeks, verified by the reassessment of the nurse and doctor of the Primary Health Care unit, the case should be discussed with e-Multi, with a view to being referred to the

Psychosocial Care Center (CAPS).
25. Consider that if the person is in a possible depressive phase of bipolar disorder, they should undergo a medical evaluation with a professional from the primary care unit and be considered for referral to the Psychosocial Care Center (CAPS) after discussing the case with the Multiprofessional team (e-Multi).
26. Consider referring the person to the Psychosocial Care Center (CAPS) in cases of severe depression with psychotic symptoms or catatonia.
27. Consider referring the person to the Psychosocial Care Centre (CAPS) in cases of previous severe depressive episodes - psychotic symptoms, attempted suicide or psychiatric hospitalization.
28. Consider referring the person to the Psychosocial Care Center (CAPS) in cases of depression and persistent suicidal ideation after initial management in the Primary Health Care unit - no improvement 12 weeks after the start of management.
29. Consider referring the person to the emergency department for immediate assessment and calling the Mobile Emergency Care Service (SAMU) if there are psychotic symptoms (persecutory behavior, guilt, talking to oneself, wandering).
30. Consider referring the person to the emergency service for immediate assessment and calling the Mobile Emergency Care Service (SAMU) if there is an association of acute suicidality (current suicidal ideas with a concrete plan, access to lethal means, visible despair and hopelessness).
31. Consider referring the person to the emergency service for immediate assessment and calling the Mobile Emergency Care Service (SAMU) if there is an association of very marked neurovegetative signs and symptoms: delayed verbal response, motor slowing, weight loss due to inappetence.

32. Consider referring to the emergency service and calling the Mobile Emergency Care Service (SAMU) in cases where there are clinical, surgical, obstetric or psychiatric complications (intoxication or substance withdrawal), agitation, aggression and/or impulsiveness.
33. Follow up on the person after they have been treated in an emergency service/are discharged from hospital as a result of being admitted to a mental health ward.

NURSING ACTIONS FOR PEOPLE IN PSYCHOLOGICAL DISTRESS WITH COMPLAINTS ASSOCIATED WITH ANXIETY DISORDER
01. Check whether the person has been worrying too much (about the future or about something that really requires attention and concern).
02. Check if the person has been feeling exhausted or more tense than usual.
03. Check if the person has been feeling very irritable or "on edge" more than usual.
04. Check if the person has difficulty relaxing.
05. Consider that if the person's answer is "yes" to at least two of the actions listed in items 01 to 04, the actions listed in items 06 to 10 must be carried out.
06. Check if the person has been sleeping badly or has difficulty sleeping.
07. Check whether the person has had a headache, neckache, shoulder pain, backache or headache.
08. Check for dizziness, cold sweats, diarrhea, tingling, stomach discomfort, churning.
09. Check if the person is worried about their health.
10. Check whether the problems reported by the person have affected their quality of life and relationships with other people.
11. Consider that if there have been 05 or more positive responses with at least 06 months of evolution, they indicate a strong risk for an anxiety disorder.
12. Offer psychoeducation to all people, their families and caregivers during the assessment or through information groups about what anxiety is and the techniques that can reduce symptoms.
13. Instruct, during individual or group psychoeducation, that in cases of anxiety attacks, the person can use diaphragmatic breathing: place one hand on the stomach and the other on the chest and only the hand on the stomach should move while breathing slowly

through the nose.
14. Instruct the person during individual or group psychoeducation to develop processes of reflection on the rationale of thoughts in cases where negative or unpleasant thoughts arise.
15. Instruct the person during individual or group psychoeducation not to try to push negative thoughts away, emphasizing that the important thing is not to give them importance and that they will disappear over time.
16. Offer people the chance to take part in Integrative and Complementary Practices (ICPs) groups.
17. Reassure the person in panic attacks (sudden and unexpected sensation of terror, associated with autonomic symptoms, particularly cardiorespiratory symptoms), informing them that the symptoms are due to an anxiety attack, unrelated to a serious medical condition with an imminent risk of death.
18. Reinforce to the person that panic attacks are only temporary (a few minutes).
19. Instruct the person in panic attacks to breathe through their nose and not through their mouth, so as not to hyperventilate.
20. Request medical assessment in cases where the person has intense and prolonged crises.
21. Consider referring the person to the Alcohol and Other Drugs Psychosocial Care Center (CAPS AD), after discussing the case with the team or at matrix support meetings, in cases where there is an association with a serious psychoactive substance use disorder.
22. Consider referring the person to the Psychosocial Care Center (CAPS), by discussing the case with the team or at matrix support meetings in cases where there is a comorbidity with bipolar disorder or psychotic symptoms.

23. Consider referring people with anxiety disorders and persistent suicidal ideation to the Psychosocial Care Center (CAPS) after initial management in Primary Health Care for 12 weeks without improvement after starting management.
24. Consider referral to the Psychosocial Care Center (CAPS), by discussing the case in the team or in matrix support meetings for refractory people - lack of response or partial response to two effective therapeutic strategies (psychotropic drugs and/or psychotherapy) for at least 8 weeks.
25. Consider referring the person to the emergency department for immediate assessment and calling the Mobile Emergency Care Service (SAMU) if the person manifests the association of acute suicidality (current suicidal ideas with a concrete plan, previous attempts, substance abuse, access to lethal means, visible despair and hopelessness).
26. Follow up on the person after they have been treated in an emergency service/are discharged from hospital as a result of being admitted to a mental health ward.

NURSING ACTIONS FOR PEOPLE IN PSYCHOLOGICAL DISTRESS WITH SUICIDAL IDEATION
01. Carry out qualified listening with the person in a private environment, without interruptions, leaving them free to expose and identify what their urgency is.
02. Use a calm, accepting, non-judgmental approach and pay attention to non-verbal expressions.
03. Trying to establish a therapeutic of trust, empathy, authenticity and respect.
04. Use the D(s) rule as a mnemonic, which includes mental disorders and affective states associated with suicide: psychic pain, despair, hopelessness, helplessness, depression, drug addiction, delirium, delirium.
05. Assess suicidal intent, checking for ideas of death: Have you ever thought that it would be better to die? What are these thoughts like? Have you thought about taking your own life? When did these thoughts start?
06. Assess suicidal intent, checking for suicidal ideation: Do suicidal thoughts persist? Do they frighten you? Can you push them away? Do you find reasons to stay alive?
07. Assessing suicidal intent, checking for a suicide plan: Have you thought about how to kill yourself? Did you find out about a method? Firearms, poisons, medication? Have you made prior arrangements?
08. Consider predisposing factors when assessing suicide risk, such as: suicide attempt, psychiatric disorders, suicide in the family, physical or sexual abuse in childhood, impulsiveness/aggressiveness, social isolation, incapacitating/incurable illnesses, despair and restlessness, recent discharge from psychiatric hospitalization. (added).
09. Consider precipitating factors when assessing suicide risk: disappointment in love, marital separation, relationship conflicts, financial collapse, job loss, humiliation/dishonor,

drunkenness, access to a lethal means.
10. During the assessment, check for protective factors against suicide by asking what the family environment is like and who they live with and how their relationship is at home.
11. During the assessment, check for protective factors against suicide by asking about relationships in the school, professional and social spheres, and belief in and attendance at religious services.
12. When formulating the risk of suicide, consider the following as low risk: never attempted suicide, suicidal ideations are fleeting and disturbing, no plans to kill one self, mental disorder present and with controlled symptoms, good adherence to treatment, life and social support.
13. When formulating suicide risk, consider the following as moderate risk: previous suicide attempt, depression or bipolar disorder, persistent ideas of suicide seen as a solution, does not have a plan on how to kill themselves, is not an impulsive person, does not abuse/depend on alcohol or other drugs, has social support.
14. When formulating suicide risk, consider as high risk: previous suicide attempt, severe depression, influence of delirium and hallucination, alcohol abuse/dependence, despair, intolerable psychological torment, no way out, definite plan to kill one self, has the means so, has already taken steps towards the suicidal act.
15. Consider using the Nurses Global Assessment Suicide Risk Scale (NGASR) to assess suicide risk stratification.
16. Promoting safety (supervision and restricting access to means of self-injury) in the event of a serious risk of suicide.
17. Help the person to see their strengths (for example, reinforce that the choice to seek help was a good one), validate their feelings and help them regain control.

18. Identify and strengthen the support network, if possible, with the person's consent.
19. Demonstrate that you accept the person's desire not to feel pain and convey your desire to support them in finding healthy alternatives for dealing with pain.
20. Involve family, friends and others in the risk assessment and treatment of suicide-related behavior, when appropriate and with the person's knowledge and consent, in order to ensure monitoring and safety.
21. Establish a follow-up plan with the person so that care can be continued, such as recognizing warning signs, identifying internal coping strategies, identifying interpersonal support, contacting significant people to help resolve the crisis; contacting health services that have a care link; reducing potential access to lethal means.
22. Consider that if there are depressive symptoms and suicidal ideation, but no plans to commit suicide, good social support and no history of psychoactive substance use, care can be maintained in the Primary Care Unit in order to monitor that suicidal ideation does not evolve into planning and attempting suicide.
23. Consider that if there are depressive symptoms and suicidal ideation, no plans to commit suicide, but a previous attempt, and/or weak social support and/or alcohol and drug use, the case should be discussed with a Multiprofessional team (e-Multi) or Psychosocial Care Center (CAPS) to assess the need for follow-up.
24. Consider that if there are severe depressive symptoms and plans to commit suicide, the support network should be informed and advised not to leave the person alone and to take them to a general hospital.
25. Consider calling the Mobile Emergency Care Service (SAMU) for transfer to a general hospital in cases where the person has ideation, plans and access to plans, lack of social support, determination to carry out the plans, psychomotor agitation
26. Consider that, once the condition has stabilized, the person's care should be

coordinated between the Primary Health Care Unit, the Multiprofessional team (e-Multi) and the Psychosocial Care Centre (CAPS) in order to monitor suicidal ideation in relation to the evolution of planning and attempts.

NURSING ACTIONS FOR PEOPLE IN PSYCHOLOGICAL DISTRESS DUE TO THE USE OF PSYCHOACTIVE SUBSTANCES
01.Carry out qualified listening to the person and their family member (when present), in a private environment).
02. Identify, during listening, the main complaint and characteristics of the suffering, life history, clinical conditions, context of drug use, seeking to build a bond.
03.Offering the person, during the listening session, access to health care and guidance to reduce the harm related to use, seeking to build a bond.
04. Use the CAGE questionnaire (Cutdown, annoyed by criticism, Guilty and Eye-opener) to detect harmful use of alcohol.
05. Use the AUDIT (Alcohol Use Disorders Identification Test) questionnaire to investigate the quantity, frequency, withdrawal symptoms associated with alcohol and associated problems.
06. Use the ASSIST (Alcohol, Smoking and Substance Involvement Screening Test) questionnaire to check whether the person has used drugs in life and in the last three months and to assess dependency.
07 - Carry out a brief intervention for people who use psychoactive substances, using the acronym FRAMES: F- Feedback- use information provided by the person about problems in their life and check for an association with substance use; R- Responsibility- negotiate with the person about goals to be achieved during treatment; A- Advice- clear recommendations for the person to change their habits; M- Menu of options- identify with the person strategies for changing their behavior; E- Empathy- show understanding to the person. S- Self- efficacy-encouraging the person about their ability to modify their behavior.

08. Suggest that the person attend other spaces in the municipality's community network, such as the Coexistence and Cooperative Center (CECCO).
09. Suggest, in cases of addiction, that the person attend self-help groups, such as Alcoholics Anonymous (AA) and Narcotics Anonymous (NA).
10. Consider that if the person is using drugs, but is not in serious psychological distress and maintains a functional relationship with the various areas of life, they should be cared for in the primary care unit and/or street clinic.
11. Consider that, in the case of a severe case, with intense suffering in which the person makes harmful use of drugs, adding to situations of clinical and social vulnerability, the person should have their care shared with the Alcohol and Other Drugs Psychosocial Care Center (CAPS AD).
12. consider referring the person to a general hospital by calling the Mobile Emergency Care Service (SAMU) in cases of acute intoxication by psychoactive substances (medication, alcohol and other drugs) with clinical repercussions and a lowered level of consciousness, and/or psychomotor agitation and/or aggression involving a risk to themselves or others.
13. Consider referring the person to a general hospital, by calling the Mobile Emergency Care Service (SAMU) in cases of harmful use of alcohol and other drugs, with agitation and/or self- or hetero-directed aggression, refractory to the approach.
14. Consider referring the person to a general hospital, by calling the Mobile Emergency Care Service (SAMU) in cases of alcohol and other drug use, which culminate in self-inflicted violence or suicidal ideation with structured planning and/or a consolidated suicide attempt in a recent episode.

15. Consider referring the person to a general hospital, by calling the Mobile Emergency Care Service (SAMU), in cases of alcohol and other drug use that culminate in an attempt to harm oneself or leave the space to carry out the act.
16. Consider referring the person to a general hospital, by calling the Mobile Emergency Care Service (SAMU) in cases of harmful use of alcohol and other drugs, delirium or withdrawal.
17. Follow up on the person after they have been treated in an emergency service/are discharged from hospital as a result of being admitted to a mental health ward.

NURSING ACTIONS TOWARDS PEOPLE IN PSYCHOLOGICAL DISTRESS AS A RESULT OF BEREAVEMENT SITUATIONS
01. Carry out qualified listening, in order to understand what is happening to the person, letting them talk about the loss.
02. Show empathy for the person's grief.
03. Encourage the person to express feelings about the loss.
04. Helping the person to identify personal coping strategies.
05. Help the person by bringing reality to what happened, correcting inadequate perceptions and showing how looking for a culprit won't change the outcome (if appropriate to the situation).
06. Reflect with the person that mourning is a natural process in the face of an important loss.
07. Recognize the following as normal characteristics of bereavement in an initial period: somatic or physical stress, preoccupation with the image of the person lost, guilt in relation to the person or circumstance of their death, hostile reactions of nonconformity.
08. Recognize signs that grief is not evolving properly: such as: the person can't speak without showing intense and recent feelings, minor events trigger an intense or disproportionate grief reaction. Mental Health in PHC: a multi-professional approach
09. Recognize other signs that grief is not evolving properly, such as: the person doesn't want to touch the "deceased's" belongings, has physical symptoms similar to those of the person who died and radical changes in lifestyle, subclinical depression and/or mania.
10. Recognize other signs that grief is not evolving properly, including: compulsion to imitate the person they have lost, self-destructive impulses, seasonal depression, phobia of illness and death, stationary or declining performance.

11. Mobilize support networks in the community that can support the person's grieving process.
12. Advise the person that bereavement has mental and physical effects, such as emptiness, tightness in the chest, lump in the throat, shortness of breath, lack of energy, muscle weakness, disbelief, sleep and appetite disorders, social isolation, dreaming about the person who has died, crying, visiting places, carrying significant objects.
13. Advise the person that there is no right or wrong way to experience grief and that sometimes they will feel very sad; other times they will feel numb; and at other times they will even be able to enjoy themselves, but that, in general, these experiences will become less intense and less frequent as time goes by.
14. Assess the person for prolonged bereavement disorder and, in this case, discuss the case in a multi-professional team (e-Multi) and, if necessary, possibly share care with the Psychosocial Care Center).
