# Supplementary material for: Construction and Validation of Nursing Actions to Integrate Mobile Care–Educational Technology to Assist Individual in Psychic Distress
Source: Int J Environ Res Public Health. 2025 Mar 13;22(3):419. doi: 10.3390/ijerph22030419 (PMC11941836; doi:10.3390/ijerph22030419)
Supplement: Supplementary file 1 [file ijerph-22-00419-s001.zip › Additional files -Table S2 - SEARCH STRATEGY.pdf]

Table S2 - Search strategies. São Paulo, Brazil, 2023.

| Database | Search Engine |
|---|---|
| APA PsycINFO | Any Field: Nurses *AND* Any Field: Distress *AND* Any Field: "Primary Health Care" *AND* Year: 2011 *To* 2022 |
| LILACS | (( mh:("Mental Health")) OR "Mental Health") AND (( mh:("Primary Health Care")) OR "Primary Health Care") AND (( mh:("Nurses")) OR Nurses)<br><br>(( mh:("Psychological Distress")) OR "Psychological Distress") AND (( mh:("Primary Health Care")) OR "Primary Health Care") AND (( mh:("Nurses")) OR Nurses) |
| WEB OF SCIENCE | Nurses AND "Psychological distress" AND "Primary health care" |
| SCIELO | (enfermagem) AND ("sofrimento mental" OR "Sofrimento psíquico") AND ("atenção primária à saúde")<br>(enfermagem) AND (sofrimento) AND ("saúde mental") AND ("atenção primária à saúde") |
| CINAHL | ((MH "Psychological Distress") OR "Psychological Distress" ) AND ( (MH "Primary Health Care") OR "Primary Health Care" ) AND ( (MH "Nurses") OR "Nurses" ) |
| AGELINE | ("Psychological Distress") AND ("Primary Health Care") AND ((MH "Nurses") OR "Nurses") |
| MEDLINE | ("Nurses"[Mesh] OR Nurses) AND ("Psychological Distress"[Mesh] OR "Psychological Distress") AND ("Primary Health Care"[Mesh] OR "Primary Health Care") |
| SCOPUS | TITLE-ABS-KEY (nurses AND "Psychological Distress" AND "Primary Health Care" ) |
| PEPSIC | enfermeiros [Todos os índices] and "Atenção Primária à Saúde" [Todos os índices] and "Saúde Mental" [Todos os índices] |
| BVS | ((mh:("Psychological Distress")) OR "Psychological Distress") AND (( mh:("Primary Health Care")) OR "Primary Health Care") AND (( mh:("Nurses")) OR Nurses) |

Source: Prepared by the authors, 2023
