# Supplementary material for: Construction and Validation of Nursing Actions to Integrate Mobile Care–Educational Technology to Assist Individual in Psychic Distress
Source: Int J Environ Res Public Health. 2025 Mar 13;22(3):419. doi: 10.3390/ijerph22030419 (PMC11941836; doi:10.3390/ijerph22030419)
Supplement: Supplementary file 1 [file ijerph-22-00419-s001.zip › Additional files -Table S3- Nacional and internacional manuals and relevant literature.pdf]

Table S3: National and international manuals and relevant literature.

**National and International Manuals**
1. Basic Care notebook, Nº34
2. World Organization of Family Doctors.
3. Manual of Interventions for mental, neurological disorders and disorders caused by the use of alcohol and other drugs in the Primary Health Care network.
4. Addressing common mental suffering in Primary Care in 7 steps.
5. Babel Mental Health Card in Primary Care.
6. Lines of Care from the Primary Care Office. Depression in adults.
7. Referral Protocols for Adult Psychiatry.
8. Lines of Care from the Primary Care Office. Anxiety disorders in adults. [22-25]
9. Suicide risk prevention: guide for health professionals.
10. Collection Quick Reference Guide, Suicide Risk Assessment and Prevention.
11. Strategic guide for the care of individuals with needs related to the consumption of alcohol and other drugs.
12. Collection Quick Reference Guide, Alcohol and other drugs.
13. Guidelines on care for mental health crises and longitudinal monitoring of cases in the Psychosocial Care Network in the city of São Paulo.
14. Nursing Protocol in Primary Care. Issue - Nursing in Mental Health Care in the Context of Primary Health Care.
15. Classification of Nursing Interventions (NIC).
16. Mental health in primary care: multi-professional approach - 1st. ed.
17. Suicide Crisis: assessment and management, 2nd. ed.
18. Nurses Global Assessment Risk Suicide.
19. AUDIT (Alcohol Use Disorders Identification Test).

20. CAGE (Cut down, Annoyed by criticism, Guilty, Eye-opener).
21. ASSIST (Alcohol, Smoking and Substance Involvement Screening Test).

Source: Author1
