# Supplementary material for: Construction and Validation of Nursing Actions to Integrate Mobile Care–Educational Technology to Assist Individual in Psychic Distress
Source: Int J Environ Res Public Health. 2025 Mar 13;22(3):419. doi: 10.3390/ijerph22030419 (PMC11941836; doi:10.3390/ijerph22030419)
Supplement: Supplementary file 1 [file ijerph-22-00419-s001.zip › Additional files -Table S4- Nursing actions in the initial assessment of people with psychological distress and De.pdf]

Table S4 - Nursing actions in the initial assessment of people with psychological distress and Depressive Disorder.

Categories and items	1st Round		Items	2nd Round	
	CVI (%)	α	Changes and/or Additions	CVI (%)	α
**A - Nursing actions in the initial assessment of individual in psychic distress**	**99**	**0,47**		**98,3**	**0,83**
01. Listen, allowing people to speak without being interrupted	1,000	0,500	01. Carry out qualified listening by asking some questions: How are you feeling? How are things at home? What brings you here? How can we help you?	1,000	-
02. Make sure there is no mental disorder, pay attention to suicidal ideation, risk of self/hetero aggression, episode of mania, change in level of consciousness	1,000	0,483	02. Make sure there is no mental disorder, pay attention to suicidal ideation, risk of self-hetero aggression, episode of mania, change in level of consciousness.	1,000	0,483
03. Explore psychosocial status, experience of illness and use techniques [a]	1,000	0,453	03. Explore, while listening, the individual's psychosocial situation, using the technique [a].	1,000	0,661
04. Demonstrate an empathetic response to their problem and use techniques [b]	1,000	0,429	04. Demonstrate, while listening, an empathetic response to your problem, using a technique [b.]	1,000	0,680
05. Perform physical examination and vital signs.	1,000	0,460	05. Perform a brief physical examination and measure blood pressure, respiratory rate and cardiac and pulmonary auscultation.	0,938	0,652

06. Evaluate the physical cause that explains symptoms and verify strategies for conducting treatment.	1,000	0,468	06. Evaluate whether there is a physical factor that could explain the symptoms mentioned and, if so, check strategies for providing care and/or referral.	1,000	-
07. Understand and explain what led the person to seek the unit [c]	1,000	0,500	07. Understand and explain what, in fact, led to the search for unity, through clarifications and recaps.	1,000	0,661
08. Contextualize suffering in the various individual, social and collective dimensions[d]	1,000	-	08. Contextualize suffering in a person's life, considering the individual, social and collective dimensions[d] .	1,000	0,612
09. Disconnect the problem as something pathological that needs to be medicalized [e.]	0,875	0,469	09. Disconnect the problem that the person presents as something only pathological, which needs to be medicalized, to a perception that psychological suffering is part of the human essence, and can be related to their experience of this suffering in their life history and social context.	1,000	-
10. Carry out psychoeducation [f.]	1,000	0,399	10. Carry out psychoeducation about psychic distress with the individual	1,000	0,611
11. Develop problem-solving skills [g].	1,000	0,294	11. Develop problem-solving skills [g.]	1,000	0,294
12. Develop a bond and ensure your trust[h] .	1,000	0,513	12. Develop a bond and ensure your trust [h.]	1,000	0,513
			13. Pay attention to the presence of signs and symptoms of physical	1,000	-

			illnesses: diabetes, hypothyroidism, lupus, Parkinson's disease, nutritional deficiency.		
			14. Identify, during listening, whether the individual uses psychoactive substances.	1,000	0,653
			15. Explore, while listening, the experience of the person's illness, using the technique[a] .	1,000	0,661
			16. Build tools with the individual to deal with psychic suffering.	0,813	0,739
**B - Nursing actions towards individuals in psychic distress with complaints associated with Depressive Disorder**	**93,4**	**0,84**		**96,4**	**0,81**
01. Identify whether in the last 2 weeks the person has felt sad, discouraged, depressed most of the days.	1,000	0,880	01. Identify whether in the last 2 weeks the person has felt sad, discouraged, depressed most of the days.	1,00	0,880
02. Identify whether, in the last 2 weeks, the person felt like no longer had any taste for anything, lost interest and pleasure in the things you used to enjoy.	1,000	-	02. Identify whether, in the last 2 weeks, the person felt like no longer had any taste for anything, lost interest and pleasure in the things you used to enjoy.	1,00	-
03. Make sure you have at least one “yes” answer to items 1 or 2, carry out actions 4 and 10.	0,938	0,875	03. Make sure you have at least one “yes” answer to items 1 or 2, carry out actions 4 and 10.	0,938	0,875

04. Ask about significant changes in appetite.	1,000	-	04. Ask the person if they have noticed a difference in their appetite over the last few days.	1,000	-
05. Check for sleep problems almost every night [i]	1,000	-	05. Check for sleep problems almost every night [i.]	1,000	-
06. Check movement slower than usual, more agitated or unable to stay still.	1,000	0,879	06. Check if the individual has been moving more slowly than usual, has no desire to carry out daily tasks or, on the contrary, has felt more agitated or unable to sit still.	0,937	0,774
07. Check tiredness most of the time, without energy, almost every day.	1,000	0,88	07. Check whether the individual has felt tired most of the time, without energy, almost every day.	1,000	0,88
08. Check feelings of worthlessness or guilt, almost every day.	1,000	0,881	08. Check whether the individual has felt a feeling of worthlessness or guilt almost every day.	1,000	0,881
09. Check for difficulty in making decisions, concentrating or memory problems almost every day.	1,000	0,878	09. Check for difficulty in making decisions, concentrating or memory problems almost every day.	1,000	0,878
10.Check for bad thoughts [j]	1,000	-	10. Check for bad thoughts [j.]	1,000	-
11. Consider the answer “yes” in actions 1 or 2 and in any of 4 to 10, a high risk of depression.	0,875	0,87	11. Consider the answer “yes” in actions 1 or 2 and in any of 4 to 10, a high risk of depression.	0,875	0,87
12. Consider 03 to 04 positive responses, mild depression.	0,813	0,862	12. Consider 03 to 04 positive responses, mild depression.	0,813	0,862
13. Consider 05 to 07 positive responses, moderate depression.	0,813	0,862	13. Consider 05 to 07 positive responses, moderate depression.	0,813	0,862

14. Consider 08 to 09 positive answers, severe depression.	0,813	0,862	14. Consider 08 to 09 positive answers, severe depression.	0,813	0,862
15. Consider that a person with depression may also present symptoms of anxiety and clinically unexplained somatic symptoms.	0,938	0,877	15. Consider that a person with depression may also present symptoms of anxiety and clinically unexplained somatic symptoms.	0,938	0,877
16. Evaluate suicide risk.	1,000	-	16. Evaluate suicide risk.	1,000	-
17. Check for bipolar affective disorder (previous mania).	0,813	0,866	17. Check if the individual has BAD by investigating previous symptoms of mania (euphoria)/depression.	0,938	0,773
18. Question the use of alcohol and other drugs.	1,000	-	18. Question the use of alcohol and other drugs.	1,000	-
19. Offer psychoeducation to the individual and family/caregivers[1]	1,000	0,881	19. Offer psychoeducation to the individual and family/caregivers: during the evaluation or through groups about what depression is, its symptoms, treatment and in cases of thoughts of self-harm, non-suicidal self-harm and suicide, the person should tell a person they trust and to go to the PHC unit.	1,000	0,78
20. Advise resuming or continuing previously enjoyable activities.	0,938	0,877	20. Advise the individual to resume or continue activities that were previously enjoyable or identify new activities.	1,000	0,768
21. Establish regular bedtimes and wake-up times.	0,938	0,878	21. Establish regular bedtimes and wake-up times.	0,938	0,878

22. Advise to maintain a regular diet despite variations in appetite.	0,938	0,877	22. Advise to maintain a regular diet despite variations in appetite.	0,938	0,877
23. Reinforce the importance of daily activities and social/community life.	0,938	0,877	23. Reinforce the importance of daily activities and social/community life.	0,938	0,877
24.Consider no improvement in 6 to 8 weeks, discuss with EFCH [m]	0,813	0,869	24. Consider no improvement in 6 to 8 weeks, discuss with EFHC [m] .	0,813	0,869
25. Consider medical evaluation in a possible depressive phase of BAD[n]	0,938	0,875	25. Consider medical evaluation in a possible depressive phase of BAD[n]	0,938	0,875
26. Consider referral to PCC[o] when severe depression with psychotic symptoms or catatonia (reference and counter-referral guide).	0,938	0,869	26. Consider referral to PCC[o] when severe depression with psychotic symptoms or catatonia (reference and counter-referral guide).	0,938	0,869
27. Consider referral to PCC if previous severe depressive episodes - psychotic symptoms, suicide attempt or psychiatric hospitalization (reference and counter-referral guide).	0,876	0,871	27. Consider referral to PCC if previous severe depressive episodes - psychotic symptoms, suicide attempt or psychiatric hospitalization (reference and counter-referral guide).	0,876	0,871
28. Consider referral to PCC in cases of depression and persistent suicidal ideation after initial management in PHC [p] (lack of improvement)	0,813	0,866	28. Consider referral to PCC in cases of depression and persistent suicidal ideation after initial management in PHC [p] (lack of improvement).	0,813	0,866
29. Consider emergency referral for immediate evaluation (referral	0,750	0,860	29. Consider referral to the emergency service for immediate	0,937	0,777

and counter-referral and Mobile Emergency Care Service (EMCS)[q] when psychotic manifestation)			evaluation and connecting EMCS ambulance, if psychotic symptoms occur (persecutory, ideas of guilt, talking to oneself, walking aimlessly).		
30. Consider referral to the emergency room for immediate assessment (referral and counter-referral and EMCS if acute suicidality)	1,000	0,877	30. Consider referral to the emergency room for immediate assessment (referral and counter-referral and EMCS if acute suicidality)	1,000	0,877
31. Consider referral to the emergency room for immediate evaluation (referral and counter-referral and EMCS if neurological signs and symptoms)	0,938	0,878	31. Consider referral to the emergency room for immediate evaluation (referral and counter-referral and EMCS if neurological signs and symptoms)	0,938	0,878
32. Consider referral to the emergency room for immediate evaluation (referral and counter-referral and EMCS if associated with clinical, surgical, obstetric or psychiatric complications)	1,000	0,874	32. Consider referral to the emergency room for immediate evaluation (referral and counter-referral and EMCS if associated with clinical, surgical, obstetric or psychiatric complications)	1,000	0,874
33. Carry out follow-up after any emergency service/hospital discharge resulting from mental health hospitalization.	1,000	0,877	33. Carry out follow-up after any emergency service/hospital discharge resulting from mental health hospitalization.	1,000	0,877

Source: Author.

CVI: Content Validation Index.

Source: Author. CVI- Content Validation Index. [I]Cronbach's alpha if item is deleted. [a] BATHE B-Background - Ask the individual how their life have been? What has been happening? A-Affect – Ask the individual how they feel about the situation; T-Troubles - Asking the person what kind of problems and difficulties this situation brings; H- Handling- Asking the individual how they have handled the situation; E-Empathy - “I realize that you are facing a (quite) difficult time and SIFE TECHNIQUE - Sentiment - the individual's feelings in relation to their problem/suffering - Ask, for example: How do you feel about (problems/suffering)? Ideas - The individual's ideas about what is wrong. For example, ask: What do you think could be causing this problem/suffering? Functioning - Effects of the disease on an individual's functioning - Ask, for example: How does this problem/suffering influence the things you do on a daily basis? Expectation - individual expectations about the care to be offered. How do you think I could help you?, respectively. [b]NURSE. (NURSE TECHNIQUE): N- Naming- “It seems to me that you are concerned about these issues”./ U- Understanding- “I understand how much this has worried you”/R- Respecting - “It must be very distressing to deal with(...)/; S- Supporting- “I would like to know how you hope I can help you”/ E- Exploring - “How are you coping with this? Ex: “What does feeling bad mean to you?” Recaps- Offer the person a summary of the information and conclude with questions.[d] Ex: “Have you ever felt this way at other times in your life? How did you handle it?”; [e] Ex: Reflect together with the person that all the suffering they experience can, unfortunately, happen to many people who are going through ;[f]Ex: Tell the individual that, given what you have discussed, you understand that they have been experiencing suffering/discomfort, sometimes with physical manifestations deeply related to difficult situations; [g]convey a message that the individual is healthy, despite the expected suffering they present, and that they have the tools to deal with this problem. (For example: I want to reinforce that you…; [h] Reinforcement - Ex: I think today's consultation was important for us to better understand their suffering and the issues related to it./ Verification - Ex: I would like to know; [i] difficulty falling asleep, waking up in the middle of the night and sleeping too much; [j]“It would be better to be dead” or “to harm yourself".[l] during the evaluation or through groups about what depression is, its symptoms, treatment and in cases of thoughts of self-harm/suicide, the person should tell someone they trust and seek help; [m] Expanded Family Health Center; [n] Bipolar Affective Disorders; [o] Psychosocial Care Center; [p] Primary Health Care; [q] Emergency mobile care service.
