# Supplementary material for: Construction and Validation of Nursing Actions to Integrate Mobile Care–Educational Technology to Assist Individual in Psychic Distress
Source: Int J Environ Res Public Health. 2025 Mar 13;22(3):419. doi: 10.3390/ijerph22030419 (PMC11941836; doi:10.3390/ijerph22030419)
Supplement: Supplementary file 1 [file ijerph-22-00419-s001.zip › Additional files- Table S5 Nursing actions for individuals in psychological distress complaints associated with an.pdf]

Table S5- Nursing actions for individuals in psychological distress: complaints associated with anxiety disorder and suicidal ideation.

Categories and items	1st Round		Items	2nd Round	
			Changes and/or Additions		
	CVI (%)	α		CVI (%)	α
**C - Nursing actions towards individuals in psychic distress with complaints associated with Anxiety Disorder**	**95,4**	**0,88**		**94,9**	**0,91**
01. Check if the individual has been worrying too much.	0,936	0,874	01. Check if the individual has been worrying too much (about the future or something that really requires attention and concern).	1,000	0,890
02. Check if the individual has been feeling exhausted, tense.	1,000	0,880	02. Check if the individual has been feeling exhausted, tense more than usual.	1,000	-
03. Check if you the individual has been feeling very irritable or has "nerve problems"?	1,000	0,882	03. Check if the individual has been feeling very irritable or has "nerve problems" more than usual.	0,937	0,899

04. Check if the individual has difficulty relaxing.	1,000	0,873	04. Check if the individual has difficulty relaxing.	1,000	0,873
05. Consider the answer “yes” in at least two of the actions in items 01 to 04, carry out actions from 06 to 10.	1,000	0,875	05. Consider the answer “yes” in at least two of the actions in items 01 to 04, carry out actions from 06 to 10.	1,000	0,875
06. Check if the individual has been sleeping poorly or has difficulty sleeping.	1,000	-	06. Check if the individual has been sleeping poorly or has difficulty sleeping.	1,000	-
07. Check if the individual has felt a headache, neck pain or discomfort in their head.	1,000	0,874	07. Check if the individual has felt a headache, neck, shoulder, back or discomfort in their head.	0,937	0,887
08. Check if the individual has been experiencing dizziness, cold sweat, diarrhea, tingling, stomach discomfort, fluster.	0,938	0,887	08. Check if the individual has been experiencing dizziness, cold sweat, diarrhea, tingling, stomach discomfort, fluster.	0,938	0,887
09. Check if the individual is concerned about their health.	0,938	0,872	09. Check if the individual is concerned about their health.	0,938	0,872

10. Check whether problems have harmed quality of life and relationships with people	1,000	-	10. Check whether problems have harmed quality of life and relationships with people	1,000	-
11. Consider that 5 or more positive responses with at least 6 months of evolution indicate a strong risk of being diagnosed with an anxiety disorder.	0,813	0,857	11. Consider that 5 or more positive responses with at least 6 months of evolution indicate a strong risk of being diagnosed with an anxiety disorder.	0,813	0,857
12. Offer psychoeducation (individuals, family members, caregivers) during assessment or through groups, information about anxiety and techniques for reducing symptoms.	1,000	0,883	12. Offer psychoeducation (individuals, family members, caregivers) during assessment or through groups, information about anxiety and techniques for reducing symptoms.	1,000	0,883
13. Instruct that in cases of anxiety attacks, the individual can use diaphragmatic breathing: place one hand on the belly and the other on the chest and only the hand on the belly should move while breathing slowly through the nose.	1,000	0,876	13. Instruct that in cases of anxiety attacks, the individual can use diaphragmatic breathing: place one hand on the belly and the other on the chest and only the hand on the belly should move while breathing slowly through the nose.	1,000	0,876
14. Instruct how to develop reflection processes on the logical basis of thoughts in	1,000	0,873	14. Instruct how to develop reflection processes on the logical basis of	1,000	0,873

cases where negative or unpleasant thoughts arise.			thoughts in cases where negative or unpleasant thoughts arise.		
15. Instruct them not to try to ward off negative thoughts, highlighting that the important thing is not to give importance to them and they will disappear over time.	0,876	0,889	15. Instruct them not to try to ward off negative thoughts, highlighting that the important thing is not to give importance to them and they will disappear over time.	0,876	0,889
16. Offer the possibility of participating in CIP[a] groups (lian-gong type).	1,000	0,881	16. Offer the possibility of participating in CIP[a] groups (lian-gong type).	1,000	0,881
17. Reassure the individual during panic attacks[b]	1,000	0,877	17.Reassure the individual during panic attacks [b]	1,000	0,877
18. Reinforce to the individual that panic attacks are temporary (a few minutes).	1,000	0,881	18. Reinforce to the individual that panic attacks are temporary (a few minutes).	1,000	0,881
19. Instruct in panic attacks, to breathe through the nose and not through the mouth (do not hyperventilate).	0,875	0,884	19. Instruct in panic attacks, to breathe through the nose and not through the mouth (do not hyperventilate).	0,875	0,884

20. Request medical evaluation in cases of intense and prolonged crises.	1,000	-	20. Request medical evaluation in cases of intense and prolonged crises.	1,000	-
21. Consider referral to PCC AD[c], after team or matrix discussion if association with psychoactive substance use disorder.	0,875	0,860	21. Consider referral to PCC AD[c], after team or matrix discussion if association with psychoactive substance use disorder.	0,875	0,860
22. Consider referral to PCC, after team or matrix discussion, if associated with comorbidity such as BAD or psychotic symptoms.	0,875	0,860	22. Consider referral to PCC, after team or matrix discussion, if associated with comorbidity such as BAD or psychotic symptoms.	0,875	0,860
23. Consider referral to PCC, after team or matrix discussion if anxiety disorder and persistent suicidal ideation, after initial treatment in PHC.	0,938	0,862	23. Consider referral to PCC, after team or matrix discussion if anxiety disorder and persistent suicidal ideation, after initial treatment in PHC.	0,938	0,862
24. Consider referral to PCC, after team or matrix discussion of refractory people (lack of or partial response to 2 effective therapeutic strategies (psychotropic drugs and/or psychotherapy).	0,875	0,860	24. Consider referral to PCC, after team or matrix discussion of refractory people (lack of or partial response to 2 effective therapeutic strategies (psychotropic drugs and/or psychotherapy).	0,875	0,860

25. Consider referral to the emergency room for immediate evaluation (referral and counter-referral and EMCS of the person who manifests the association of acute suicidality (current ideas of suicide with a concrete plan, previous attempts, substance abuse, access to lethal means, visible despair and hopelessness).	0,938	0,869	25. Consider referral to the emergency room for immediate evaluation (referral and counter-referral and EMCS of the person who manifests the association of acute suicidality (current ideas of suicide with a concrete plan, previous attempts, substance abuse, access to lethal means, visible despair and hopelessness).	0,938	0,869
26. Carry out follow-up after any emergency service/hospital discharge resulting from mental health hospitalization.	0,938	0,870	26. Carry out follow-up after any emergency service/hospital discharge resulting from mental health hospitalization.	0,938	0,870
**D - Nursing actions towards individuals in psychic distress with Suicidal Ideation**	**96**	**0,71**		**95,2**	**0,89**
01. Do the listening in a private environment, without interruptions.	1,000	-	01. Carry out qualified listening with the individual in a private environment, without interruptions, leaving them free to express and identify their urgency.	1,000	

02. Use a calm, accepting, non-judgmental approach and pay attention to non-verbal expressions.	1,000	-	02. Use a calm, accepting, non-judgmental approach and pay attention to non-verbal expressions.	1,000		-
03. Seek to establish a therapeutic relationship of trust, empathy, authenticity and respect.	1,000	-	03. Seek to establish a therapeutic relationship of trust, empathy, authenticity and respect.	1,000		-
04. Welcome in an integral, careful and individualized way, encouraging recognition and expression of feelings, according to their needs and anxieties, favoring the understanding of what is happening.	1,000	-	04. Welcome in an integral, careful and individualized way, encouraging recognition and expression of feelings, according to their needs and anxieties, favoring the understanding of what is happening.	1,000		-
05. Evaluate the risk of suicide [d].	0,938	0,814	05. Consider applying the NGASR in evaluating suicide risk stratification.	0,937	0,915	-
06. Promote safety (supervision and restriction of access to means of self-harm) in cases of serious risk of suicide.	1,000	-	06. Promote safety (supervision and restriction of access to means of self-harm) in cases of serious risk of suicide.	1,000		-
07. Help to see strengths[e].	0,938	0,757	07. Help to see strengths [e].	0,938		0,757

08. Strengthen and identify the support network, with the individual’s consent.	0,938	0,714	08. Strengthen and identify the support network, with the individual’s consent.	0,938		0,714
09. Demonstrate that you accept the individual's desire not to feel pain and convey the desire to support them in finding healthy alternatives on how to cope.	1,000	0,778	09. Demonstrate that you accept the individual's desire not to feel pain and convey the desire to support them in finding healthy alternatives on how to cope.	1,000		0,778
10. Think together about therapeutic paths and healthy ways to deal with suffering.	1,000	0,794	10. Think together about therapeutic paths and healthy ways to deal with suffering.	1,000		0,794
11. Involve family, friends, and others in risk evaluate and treatment of suicidal behavior, with consent to ensure monitoring and safety.	1,000	-	11. Involve family, friends, and others in risk evaluate and treatment of suicidal behavior, with consent to ensure monitoring and safety.	1,000		-
12. Establish care plan and continued monitoring[f]	1,000	-	12. Establish care plan and continued monitoring [f.]	1,000		-
13. Consider that, if there are severe depressive symptoms and suicide plans, refer	0,876	0,725	13. Consider that, if there are severe depressive symptoms and plans to commit suicide, the support network must be informed and instructed not to	0,937	0,931	-

to a general hospital for evaluation and need for hospitalization or to PCC for follow-up.			leave the person alone and take them to the general hospital.			
14. Contact EFHC and PCC at the time of service to discuss the case, if there is any question.	0,876	0,673	14. Contact EFHC and PCC at the time of service to discuss the case, if there is any question.	0,876		0,673
15. Consider that, after stabilization of the condition, care should be maintained in PHC, with monitoring of suicidal ideation, and progression to planning and attempt.	0,875	0,650	15. Consider that, after stabilization of the condition, care must be coordinated between PHC, Multidisciplinary team (e-Multi) and PCC in order to monitor suicidal ideation in relation to the progression to planning and attempt.	0,812	0,928	-
			16. Use the D(s) rule as a mnemonic, which includes mental disorders and affective states associated with suicide: (psychic) disease, despair, dismay, destitution, depression, (chemical) dependency, delusion, delirium.	0,875	0,921	
			17. Evaluate suicidal intent, checking for suicidal ideation: Do suicidal thoughts persist? Do they scare you?	0,937	0,915	

Can you keep them away? Do you find reasons to stay alive?		
18. Evaluate suicidal intent, checking suicidal plans: Have you thought about how to kill yourself? Did you find out about a method? Firearms, poisons, medicines? Have you already taken prior action?	0,937	0,915
19. Consider predisposing factors when evaluating the risk of suicide, such as: attempted suicide, psychiatric disorders, suicide in the family, physical or sexual abuse in childhood, impulsivity/aggressiveness, social isolation, disabling/incurable diseases, despair and restlessness, recent discharge from hospitalization psychiatric.	0,937	0,917
20. When evaluating the risk of suicide, consider precipitating factors: romantic disappointment, marital separation, relational conflicts, financial collapse, loss of job,	0,937	0,915

humiliation/dishonor, drunkenness, access to a lethal means.		
21. During the evaluation, check the protective factors against suicide, asking about the family environment and who do the person live with and what their relationship is like at home.	0,937	0,915
22. During the assessment, check protective factors against suicide, asking about relationships in the school, professional and social spheres, beliefs and attendance at religious services.	0,937	0,915
23. When formulating the risk of suicide, consider low risk: never attempted suicide, thoughts of suicide are temporary and disturbing, do not plan how to kill themselves, mental disorder present and with controlled symptoms, good adherence to treatment, having life and social support.	0,937	0,917

24. When formulating suicide risk, consider as moderate risk: previous suicide attempt, depression or bipolar disorder, persistent thoughts of suicide seen as a solution, the individual does not have a plan on how to kill him/ herself, the individual is not an impulsive person, the individual does not abuse/depend on alcohol and other drugs, the individual has social support.	0,937	0,917
25. When formulating the risk of suicide, consider as high risk: previous suicide attempt, severe depression, influence of delirium and hallucination, alcohol abuse/dependence, despair, intolerable psychic torment, the individual sees no way out, the individual has defined plan to kill him/ herself, the individual has means of how to do it, the individual has already taken steps to commit suicide.	0,937	0,915
26. Consider that, if there are depressive symptoms and suicidal ideation, but without plans to commit	1	-

	suicide, with good social support and without a history of psychoactive substance use, care can be maintained in the Primary Care Unit in order to monitor whether suicidal ideation evolves into planning and trying.		
	27. Consider that, if there are depressive symptoms and suicidal ideation, without plans to commit suicide, but with a previous attempt, and/or weak social support and/or use of alcohol and drugs, the case should be discussed with a Multidisciplinary team (e-Multi) or with a PCC to assess the need for monitoring/reception.	0,937	0,931
	28. Consider connecting the EMCS for transfer to the general hospital, in cases where the individual presents ideation, plans and access to plans, lack of social support, determination to carry out plans, psychomotor agitation.	0,937	0,931

	29. Consider that, after stabilization of the condition, the individual's care must be coordinated between the Primary Health Care Unit, the Multidisciplinary team (e-Multi) and the PCC in order to monitor suicidal ideation in relation to evolution to planning and trying.	1	-
	30. Evaluate suicidal intent, checking ideas of death: Have you ever thought it would be better to die? What are these thoughts like? Did you think about taking your own life? When did these thoughts start?	1	0,933

Source: Author.

CVI- Content Validation Index.

[1]Cronbach's alpha if item is deleted

[a] Complementary Integrative Practices;

[b] sudden and unexpected feeling of terror, associated with autonomic symptoms, particularly cardiorespiratory ones, informing that the symptoms are coming from an anxiety attack, unrelated to a serious clinical condition with a risk of imminent death;

[c]Psychosocial Care Center for Alcohol and Other Drugs;

[d]Apply the Nurses Global Assessment Risk Suicide Scale (NGASR);

[e]reinforce that the choice to seek help was a good one, validate her feelings, and help her regain control;

[f] recognition of warning signs; identification of internal coping strategies; identification of interpersonal supports as a means of distracting unpleasant thoughts or impulses; contact with significant people to help resolve the crisis; contact with health services that have a care link; reduce potential access to lethal means.
